# Association between prescribed antidepressant medication and skin cancer (melanoma, basal cell carcinoma and cutaneous squamous cell carcinoma) risk: a systematic review and meta-analysis

**DOI:** 10.1093/skinhd/vzag028

**Published:** 2026-04-17

**Authors:** Tabassum Rahman, Mikolaj Swiderski, Zoe C Venables, Zenas Z N Yiu, Lesley E Rhodes, Neil Nixon, Sonia Gran

**Affiliations:** School of Medicine, University of Nottingham, Nottingham, UK; Centre of Evidence Based Dermatology, School of Medicine, University of Nottingham, Nottingham, UK; Department of Dermatology, Norfolk and Norwich University Hospital, Norwich, UK; Norwich Medical School, University of East Anglia, Norwich, UK; Division of Musculoskeletal and Dermatological Sciences, School of Biological Sciences, Faculty of Biology, Medicine and Health, NIHR Manchester Biomedical Research Centre, University of Manchester, Manchester, UK; Department of Dermatology, Salford Royal Hospital, Northern Care Alliance NHS Foundation Trust, Manchester Academic Health Science Centre, Manchester, UK; Division of Musculoskeletal and Dermatological Sciences, School of Biological Sciences, Faculty of Biology, Medicine and Health, NIHR Manchester Biomedical Research Centre, University of Manchester, Manchester, UK; Department of Dermatology, Salford Royal Hospital, Northern Care Alliance NHS Foundation Trust, Manchester Academic Health Science Centre, Manchester, UK; School of Medicine, University of Nottingham, Nottingham, UK; Mental Health and Clinical Neurosciences, School of Medicine, University of Nottingham, Nottingham, UK; Centre of Evidence Based Dermatology, School of Medicine, University of Nottingham, Nottingham, UK

## Abstract

**Background:**

Certain antidepressants act as photosensitizers and may affect skin cancer risk; however, no evidence synthesis has been conducted. With the rising global incidence of skin cancer and widespread antidepressant use, clarifying any potential association is essential for public health.

**Objectives:**

To assess the association between prescribed antidepressants and skin cancer (basal cell carcinoma, cutaneous squamous cell carcinoma and melanoma) risk.

**Methods:**

The protocol was registered on PROSPERO. MEDLINE and Embase were searched on 5 October 2025. Titles, abstracts and full texts were screened, and the methodologies appraised by two independent reviewers. The Mantel–Haenszel method (random-effects model) was applied to pool odds ratios (ORs), and the inverse variance method was used for rate ratios (RRs); heterogeneity was assessed using *I*^2^. The GRADE (Grading of Recommendations Assessment, Development, and Evaluation) framework assessed the certainty of evidence. Subgroup analyses included a comparison of skin cancer risk between selective serotonin reuptake inhibitors (SSRIs) and non-SSRIs.

**Results:**

Ten studies were included, five of which contributed to the meta-analysis. The association between antidepressant use and skin cancer risk was not statistically significant [RR 0.83, 95% confidence interval (CI) 0.60–1.16 (*P* = 0.28, *I*^2^ = 87%); OR 0.93, 95% CI 0.85–1.02 (*P* = 0.12, *I*^2^ = 69%)]. The subgroup analysis results for those who did and did not use SSRIs were similar and showed no statistically significant associations with skin cancer risk. The certainty of evidence was very low.

**Conclusions:**

No statistically significant association was found between antidepressant use and skin cancer risk. Further high-quality research considering important confounders such as skin type, skin cancer type, sun exposure-related behaviour and sufficient follow-up is needed to confirm these findings.

## Teaser

The incidence of skin cancer is rising. Antidepressants are known to act as photosensitizers, potentially increasing susceptibility to ultraviolet radiation and the risk of carcinogenesis. Conversely, experimental models suggest that certain antidepressants may have anticarcinogenic properties.

What is already known about this topic?

- Some antidepressants have photosensitizing effects, increasing susceptibility to ultraviolet radiation and potentially elevating skin cancer risk.
- Preclinical studies suggest some antidepressants may exert pro- and anticarcinogenic effects.

What does this study add?

- We present a comprehensive synthesis of the evidence on the association between antidepressant use and skin cancer.
- Overall, there is no evidence of a statistically significant association between antidepressant use and skin cancer.
- Further research considering important confounders such as skin type, skin cancer type, sun exposure-related behaviour and sufficient follow-up is needed.

As incidence rates are rising globally, skin cancer is a growing public health concern. In the UK, melanoma incidence has increased by 147% since the 1990s.^1^ Between 2020 and 2040, cases of skin cancer are projected to rise by 34%, driven mainly by a 42% increase in keratinocyte cancers, including basal cell carcinoma (BCC) and cutaneous squamous cell carcinoma (cSCC).^2^ These figures show the growing scale of the issue and highlight the need to identify and address modifiable risk factors driving this trend.

Studies indicate that photosensitizing medications can increase skin sensitivity by generating reactive oxygen species (ROS) upon activation by ultraviolet (UV) radiation (UVR). These ROS can damage cellular lipids, proteins and DNA, potentially triggering the development of skin cancer.^3,4^ Although photosensitivity has been seen with antidepressants like tricyclic antidepressants (TCAs) and selective serotonin reuptake inhibitors (SSRIs), their link to skin cancer risk remains unclear.^3,5^ Therefore, more research is needed to help people make informed decisions.

Previous studies investigating the effects of antidepressants on melanoma progression in rodent models have produced conflicting findings. *In vivo* experiments have linked treatment with TCAs like amitriptyline and SSRIs like fluoxetine to increased melanoma mortality and metastasis in animal models.^6,7^ In contrast, other studies suggest that TCAs may exert antineoplastic effects on melanoma cells,^8^ and intraperitoneal fluoxetine demonstrated significant inhibition of melanoma growth.^9^

A literature review has reported cytotoxic effects of sertraline on melanoma cells; however, it addressed various cancer types broadly rather than focusing specifically on skin cancer.^10^ To date, no systematic review has comprehensively assessed the association between antidepressant use and skin cancer risk. Given the significant increase in antidepressant prescriptions, which rose in England from 18.4 million in 1998 to 70.9 million in 2018,^11^ synthesizing the available evidence is essential to inform public health and clinical practice. This study addresses that gap by conducting the first systematic review and meta-analysis to evaluate the association between prescribed antidepressants and skin cancer (BCC, cSCC and melanoma) risk across diverse geographical regions.

## Materials and methods

A systematic review was conducted following PRISMA guidelines.^12^ The protocol was registered with PROSPERO (CRD42024524559) on 18 March 2024.

### Search strategy

Literature searches were conducted on 5 October 2025 in OVID MEDLINE and OVID Embase using a strategy described in Appendix S1 (see Supporting Information). Grey literature was searched via ProQuest Dissertations & Theses (https://about.proquest.com/en/products-services/pqdtglobal/). Searches were not limited by publication date, language or geographical location. Reference lists were screened, Google Scholar was used to identify citing articles and trial registries were examined for additional studies. An information specialist contributed to the development of the search strategy.

### Inclusion criteria

#### Study population

Adults eligible for antidepressant prescription, with no restrictions on sex, location or publication year, were included.

#### Exposure

Any prescribed antidepressant medication, regardless of class, dose, duration or indication for use, was included.

#### Control

Comparators included adults from the general population who had not used prescription antidepressants during the study period. For analyses comparing which antidepressant is most associated with skin cancer, users of one class were compared with users of other classes as a separate exposure group. In case–control studies, cases were individuals with skin cancer and controls were those without.

#### Outcome

The outcome of interest was the onset of skin cancer (melanoma, cSCC and BCC). Studies using routinely collected healthcare databases were eligible for inclusion if the diagnoses of skin cancer was made by clinicians. For nondatabase studies, histopathological confirmation of the skin cancer diagnosis was required.

### Study design

Studies with observational designs (cross-sectional, case–control and cohort), randomized controlled trials (RCTs) and case series with ≥30 participants (sufficient for hypothesis testing) were included. Reviews, case reports, smaller case series (<30 participants) and animal studies were excluded.

### Study selection

All references were imported into Rayyan (https://www.rayyan.ai/) and duplicates were removed. To ensure consistency, each article was screened by two independent reviewers at the title and abstract and full-text stages (either T.R. and S.G., or T.R. and M.S.), with any discrepancies resolved through discussion and consensus.

### Data extraction

Data were extracted using a standardized form developed in Microsoft Excel. Study characteristics, outcome measures and exposure variables were collected. The primary researcher (T.R.) conducted the data extraction.

### Quality assessment

Methodological quality and risk of bias in the included observational studies were evaluated using the Risk Of Bias In Non-randomized Studies – of Exposures (ROBINS-E) tool.^13^ The overall risk of bias for each study was determined by the highest-risk domain, ensuring transparency and reproducibility. The certainty of evidence for each outcome was graded using the GRADE framework (https://book.gradepro.org/). Each study was independently assessed by two reviewers (T.R. and S.G., or T.R. and M.S.), with disagreements resolved by consensus.

In addition, study quality was further categorized using predefined protocol criteria: (i) reliable exposure and outcome assessment (e.g. medical records and prescription data); (ii) sufficient antidepressant exposure (case–control: ≥1 year; cohort or RCT: ≥80% sample retention and ≥1-year follow-up); and (iii) adjustment for key confounders. Studies meeting all three criteria were rated high quality; those meeting none, low quality; and those meeting some, moderate quality.

### Data analysis

A narrative synthesis was used to systematically summarize findings across included studies. Two primary meta-analyses were conducted: one for studies reporting odds ratios (ORs) and another for those reporting rate ratios (RRs). Meta-analyses were performed in RevMan (version 9.6; https://revman.cochrane.org/myReviews). The Mantel–Haenszel method (random effects model) was applied to pool ORs, and the inverse variance method for RRs. Heterogeneity was estimated using the DerSimonian and Laird method, with Wald-type confidence intervals (CIs) and *I*^2^ statistics to quantify heterogeneity.

A sensitivity analysis was conducted using melanoma-only studies among those that reported RRs. Due to data limitations, a separate meta-analysis for nonmelanoma skin cancer (NMSC) only was not feasible, as only one study reported nonmelanoma outcomes; therefore, comparisons between melanoma and nonmelanoma risk could not be performed.

Subgroup analyses compared skin cancer risk between SSRIs and non-SSRIs, repeated this comparison in melanoma-only studies and assessed skin cancer risk associated with antidepressant use by defined daily dose (DDD) quartiles compared with those who did not use antidepressants (henceforth ‘nonusers’). DDD quartiles were defined as Q0 (nonusers, DDD 0), Q1 (DDD 1–91), Q2 (DDD 92–365), Q3 (DDD 366–1460) and Q4 (DDD ≥1461), reflecting increasing antidepressant exposure to assess dose–response with skin cancer risk. Planned subgroup analyses (age, ethnicity, comorbidities and drug classes) were not conducted due to insufficient data.

## Results

### Study selection

**Figure 1:**
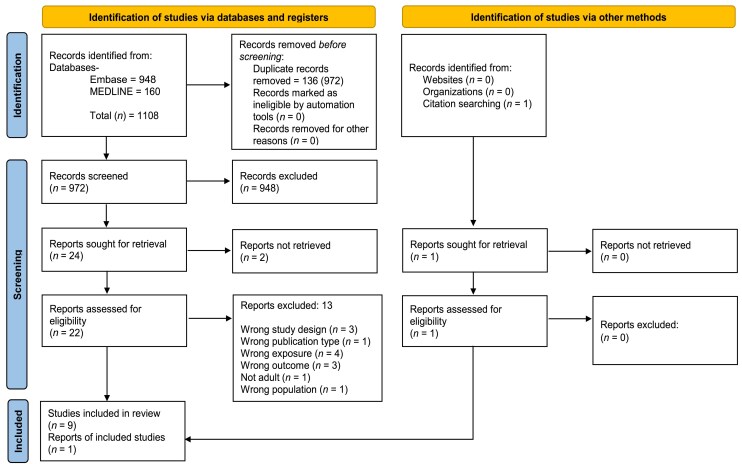
PRISMA flow diagram summarizing the search results and study selection. This figure is adapted from the PRISMA 2020 flow diagram, distributed under the terms of the Creative Commons Attribution License (CC BY 4.0).

The literature search conducted on 5 October 2025 identified a total of 1108 records (Figure 1). Following title and abstract screening, 22 full-text articles were assessed for eligibility, of which 10 met the inclusion criteria.^14–23^ Of these, five studies were eligible for inclusion in the meta-analyses,^14–17,23^ while the other five were synthesized narratively.^18–22^ Reasons for study exclusion and noninclusion in the meta-analysis are provided in Appendix S2 (see Supporting Information).

### Study characteristics

Two of the 10 included studies were of a cohort design;^22,23^ the other eight were case–control studies (Tables 1, 2).^14–21^ Geographical settings included Norway, Spain, Sweden, the UK, the USA and Finland, although none reported participants’ ethnicities. All studies were in English and included both sexes. Six studies assessed melanoma, two assessed cSCC, one assessed BCC, and one included melanoma and NMSC. Participant age ranged from 15 to 91 years, with a mean age between 45 and 75 years; participants in disease groups were often slightly older than those in control groups. Study populations varied widely in size, from small cohorts of 7–73 participants to large administrative datasets including >200 000 participants.

**Table 1:** Characteristics of the included studies: case–control studies

Study	Geographical setting and study period	Type of antidepressant (s)	Sample size (skin cancer outcome)		Control sample size (no outcome)		Outcome
			Total (*n*)	Exposed (*n*)	Total (*n*)	Exposed (*n*)	
Berge *et al*. (2020)^14^	Norway, 2004–2015	SSRI, TCA, other, mixed	12 099	1914	118 467	20 644	Cutaneous melanoma
Berge *et al*. (2020)^15^	Norway, 2004–2015	NR	12 106	1870	118 564	20 243	Cutaneous melanoma
Navarro-Bielsa *et al*. (2023)^19^	Spain, 2020–2022	NR	62	13	126	8	cSCC
Almagro *et al*. (2024)^20^	Spain, 2020–2022	NR	119	13	127	8	BCC
Navarro-Bielsa *et al*. (2025)^21^	Spain, 2020–2022	NR	73	12	126	8	Melanoma
Boursi *et al*. (2015)^16^	UK, 1995–2013	SSRI, SNRI, TCA	9226	1936	36 346	7798	Melanoma
Weiss *et al*. (1998)^18^	Oregon, USA, 1988–1994	NR	285	NR	NR	NR	Melanoma
Westerdahl *et al*. (1996)^17^	South Sweden (1988–1990)	Tri- or tetracyclic	400	11	640	13	Melanoma

BCC, basal cell carcinoma; cSCC, cutaneous squamous cell carcinoma; NR, not reported by study authors (data unavailable); SNRI, serotonin–norepinephrine reuptake inhibitor; SSRI, selective serotonin reuptake inhibitor; TCA, tricyclic antidepressant.

**Table 2:** Characteristics of the included studies: cohort studies

Study	Geographical setting and study period	Type of antidepressant(s) + control group	Sample size (exposed), *n*	Total cases (skin cancer), *n*	Cases (skin cancer outcome + exposed), *n*	Outcome
Haukka *et al*. (2009)^23^	Finland, 1998–2005	SSRI or non-SSRI vs. nonusers	SSRI = 204 070; non-SSRI = 116 616; both = 97 902	Melanoma = 623; nonmelanoma = 542	Melanoma = 280; nonmelanoma = 335	Melanoma and nonmelanoma
Knuutila *et al*. (2024)^22^	Finland (Southwest), 1994–2012	SSRI/SNRI vs. nonusers (control = AK or cSCCIS without cSCC development)	7	51	NR	cSCC

AK, actinic keratosis; cSCC, cutaneous squamous cell carcinoma; cSCCIS, cSCC *in situ*; NR, not reported by study authors (data unavailable); SNRI, serotonin–norepinephrine reuptake inhibitor; SSRI, selective serotonin reuptake inhibitor.

All included studies reported antidepressant exposure, with four studies further classifying antidepressants by type, while the remaining studies reported antidepressant use without further specification. Three studies reported antidepressant exposure in combination with hypnotics,^19–21^ and one reported exposure in combination with antihistamines.^18^ Knuutila *et al*. included patients with precancerous lesions (actinic keratosis or cSCC *in situ*), to evaluate subsequent cSCC development.^22^ Additional methodological details including exposure durations, follow-up periods, control types and age distributions per study are provided in Appendix S3 (see Supporting Information).

### Quality assessment

The detailed domain-level results of ROBINS-E are provided in Appendix S4 (see Supporting Information). Of the 10 included studies, 6 were rated as being at high risk of bias,^17–22^ and 4 as being at moderate risk.^14–16,23^ Confounding was the most common bias, as many studies did not adjust for key risk factors, including individual UV exposure, lifestyle behaviours, hormone use or socioeconomic status. Some case–control studies also exhibited potential selection bias due to companion or restricted population controls. Risk from exposure classification and deviations from intended exposures was generally low to moderate, except for Haukka *et al*.,^23^ where variable adherence to antidepressant prescriptions increased risk. The reporting of missing data was often unclear or at moderate risk of bias, whereas outcome measurement and selection of reported results were consistently at low risk of bias.

Only the study by Boursi *et al*. met all the predefined criteria, reliable exposure and outcome assessment, adequate exposure duration and comprehensive confounder adjustment, and was rated as high quality.^16^ The studies by Berge *et al*., Haukka *et al*. and Knuutila *et al*. were rated as moderate quality:^14,15,22,23^ they used validated registry or medical record data and had adequate observation periods but had incomplete confounder adjustment. The other five studies were rated low quality due to unclear or self-reported exposure, short or unverified exposure periods, and insufficient confounder control.^17–21^

Using the GRADE framework, the initial certainty was low, because all studies were observational. Further downgrades were applied for high risk of bias, inconsistent findings, and imprecision from wide CIs and small sample sizes. Therefore, the overall certainty of evidence was very low.

### Primary meta-analyses

**Figure 2:**
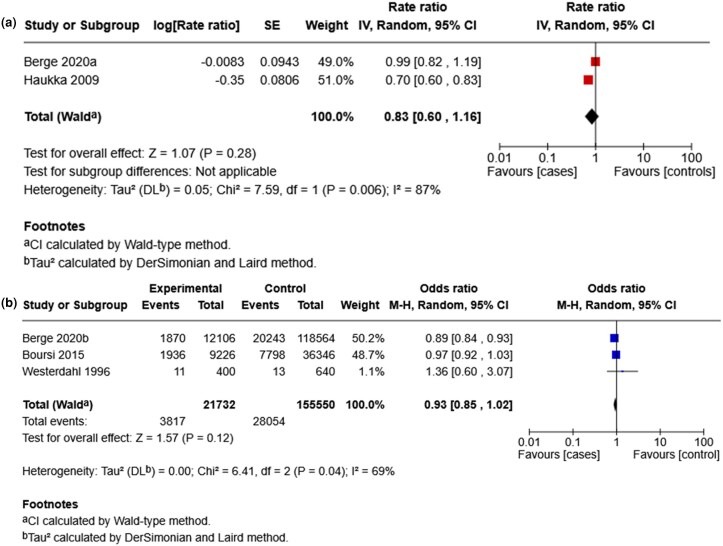
Forest plot of (a) antidepressant use and skin cancer risk (rate ratio) and (b) antidepressant use and skin cancer (odds ratio). CI, confidence interval.

To account for variation in the effect measures reported across the included studies, separate meta-analyses were conducted for studies that reported RR and OR (Figure 2). Cases of melanoma and NMSC were combined as overall skin cancer.

The pooled adjusted RR was 0.83 (95% CI 0.60–1.16), indicating no statistically significant association between antidepressant use and skin cancer risk (*Z* = 1.07, *P* = 0.28). Substantial heterogeneity was found (*I*^2^ = 87%, *P* = 0.006), suggesting considerable variability in effect estimates.

Similarly, the pooled adjusted OR was 0.93 (95% CI 0.85–1.02), indicating no evidence of a difference in the odds of skin cancer among patients who used antidepressants compared with those who did not (*Z* = 1.57, *P* = 0.12). Heterogeneity was moderate (*I*^2^ = 69%, *P* = 0.04). Individual study ORs ranged from 0.89 to 1.36, reflecting variability across studies.

### Sensitivity analysis

**Figure 3:**
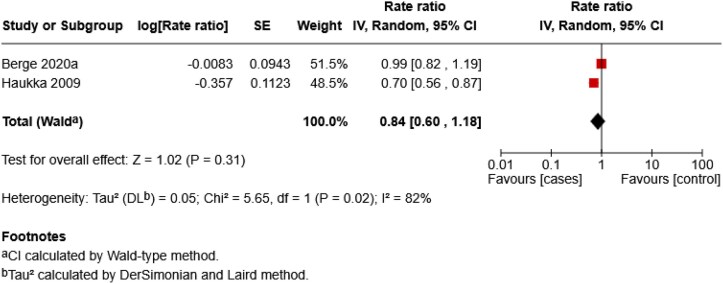
Sensitivity analysis of antidepressant use and melanoma risk (rate ratio). CI, confidence interval.

A sensitivity analysis restricted to melanoma-only studies was conducted to assess whether the inclusion of NMSC cases influenced the main results. For the OR meta-analysis, all included studies were already melanoma-specific, so the pooled estimate remained unchanged. For the RR meta-analysis, two studies reported melanoma-specific outcomes, yielding a pooled RR of 0.84 (95% CI 0.60–1.18; *Z* = 1.02, *P* = 0.31), with substantial heterogeneity (*I*^2^ = 82%, *P* = 0.02) (Figure 3).

### Subgroup analyses

**Figure 4:**
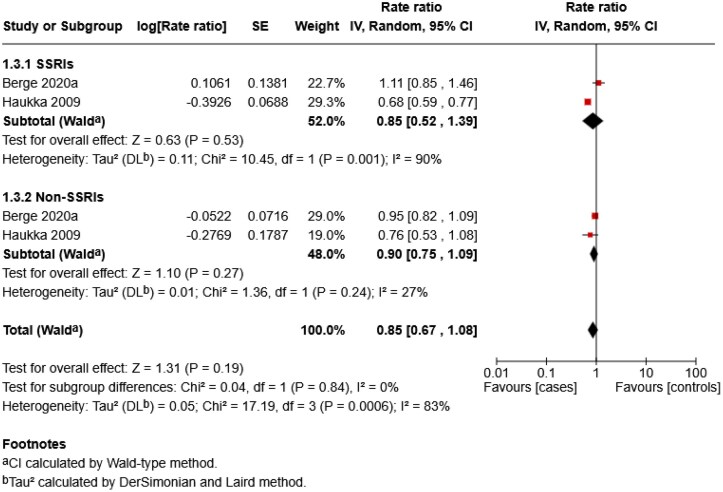
Forest plot comparing the individual effects of selective serotonin reuptake inhibitor (SSRI) and non-SSRI antidepressants on skin cancer risk. CI, confidence interval.

**Figure 5:**
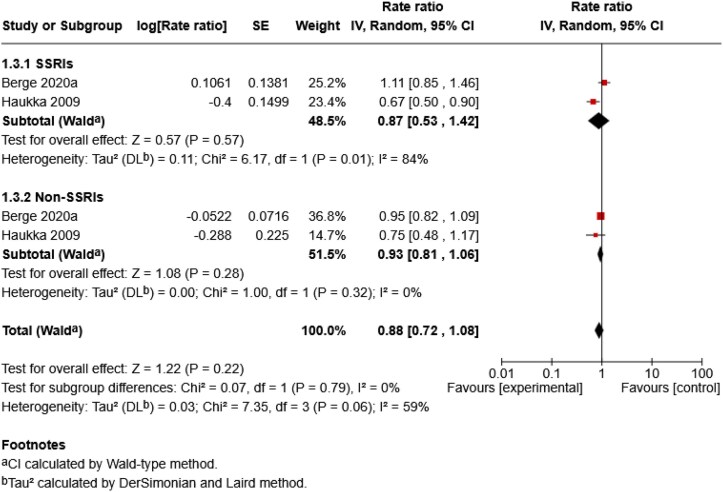
Forest plot comparing the individual effects of selective serotonin reuptake inhibitor (SSRI) and non-SSRI antidepressants on melanoma risk. CI, confidence interval.

**Figure 6:**
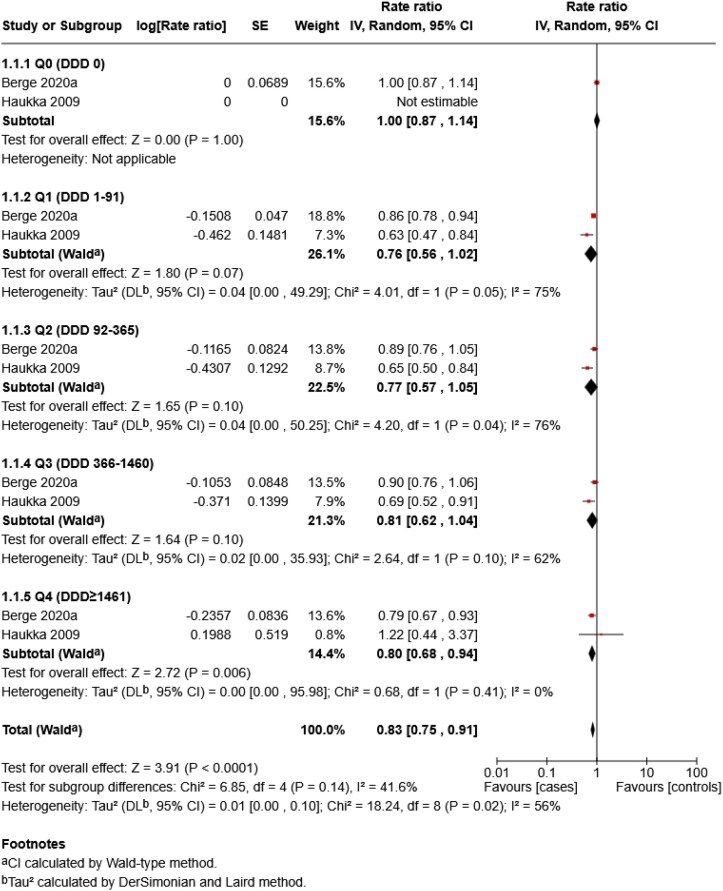
Forest plot of skin cancer risk by antidepressant defined daily dose (DDD) quartiles vs. those who did not use antidepressants (nonusers). CI, confidence interval.

Three analyses were conducted. The first analysis compared skin cancer risk between SSRIs and non-SSRIs (Figure 4). The second repeated this comparison restricted to melanoma-only studies (Figure 5). The third assessed overall skin cancer risk across quartiles of cumulative antidepressant exposure, measured in DDD, compared with nonusers (Figure 6). Only two studies contributed to the first two analyses. One classified antidepressants as SSRIs and non-SSRIs, while the other classified them as SSRIs, TCAs, mixed and other. For consistency, TCAs, mixed and other were grouped as non-SSRIs. Although monoamine oxidase inhibitors (MAOIs) were included in the search, no study reported separate data on them, so they were not included in the non-SSRI group. For the third analysis, all antidepressant types were grouped as skin cancer.

The meta-analysis showed no statistically significant association between SSRI use and skin cancer risk (RR 0.85, 95% CI 0.52–1.39; *P* = 0.53), with substantial heterogeneity (*I*^2^ = 90%, *P* = 0.001). Similarly, non-SSRIs showed no statistically significant association (RR 0.90, 95% CI 0.75–1.09; *P* = 0.27), with low heterogeneity (*I*^2^ = 27%, *P* = 0.24). No statistically significant difference was found between the SSRI and non-SSRI subgroups (*P* = 0.84, *I*^2^ = 0%).

In the melanoma-only subgroup, SSRIs showed no statistically significant association with melanoma risk (RR 0.87, 95% CI 0.53–1.42; *Z* = 0.57, *P* = 0.57), with substantial heterogeneity (*I*^2^ = 84%, *P* = 0.01). Non-SSRIs also showed no statistically significant association (RR 0.93, 95% CI 0.81–1.06; *P* = 0.28), with low heterogeneity (*I*^2^ = 0%, *P* = 0.32). No statistically significant difference was detected between the SSRI and non-SSRI subgroups (*P* = 0.79; *I*^2^ = 0%).

Dose-based analysis found that antidepressant use was associated with a lower skin cancer risk compared with nonuse (overall RR 0.83, 95% CI 0.75–0.91; *P* < 0.001). A protective trend was observed across all dose quartiles. The highest exposure group (Q4) showed a statistically significant risk reduction (RR 0.80, 95% CI 0.68–0.94; *P* = 0.006). Although the lower exposure quartiles (Q1–Q3) showed a trend towards a protective effect, their pooled estimates were not statistically significant. Moderate heterogeneity was observed overall (*I*^2^ = 56%), particularly in the lower dose groups (*I*^2^ = 75–76% in Q1 and Q2), whereas no heterogeneity was present in Q4 (*I*^2^ = 0%).

## Discussion

Our review did not find a statistically significant link between antidepressant use and skin cancer risk. A melanoma-only sensitivity analysis, restricting the primary analysis to melanoma studies, yielded nearly identical results to the primary overall skin cancer RR analysis, indicating that including cases of NMSC did not materially affect the pooled estimates of overall skin cancer.

Subgroup analyses showed that the type of antidepressant, whether SSRI or non-SSRI, did not seem to have a major influence on skin cancer risk. Melanoma-only SSRI vs. non-SSRI results were consistent with the overall skin cancer subgroup analysis. The dose–response analysis showed a significant overall reduction in skin cancer risk among patients who use antidepressants compared with those who do not, although no clear dose-dependent trend was found.

Although some variation was seen across studies, probably due to differences in study design or population characteristics, the general consistency in the direction of results suggests that antidepressant use is unlikely to increase skin cancer risk and might actually be linked to a small reduction in risk.

The lack of increased risk of skin cancer found herein aligns with a number of experimental studies that have suggested potential protective effects of antidepressants against skin cancer. Parker *et al*. reported that TCAs exert anticancer effects on melanoma cells by affecting mitochondrial membranes and disrupting lysosomes.^8^ Similarly, fluoxetine has been shown to reduce systemic oxidative stress in melanoma-bearing mice, probably by enhancing host antioxidant defences through increased activity of enzymes such as superoxide dismutase, catalase and glutathione peroxidase.^24^

Moreover, a meta-analysis found that fluoxetine reduced systemic proinflammatory cytokines, including interleukin-6 and tumour necrosis factor-α, in patients with depression, indicating immune-modulatory effects.^25^ As UVB radiation-induced skin cancers exploit immunosuppressive cytokines to evade immune detection, it is plausible that antidepressant-mediated immune modulation could contribute to a protective effect by enhancing host immune surveillance of UV-damaged skin cells.^26^ Niu *et al*. found that venlafaxine may induce apoptosis in melanoma cells,^27^ and the review by Uversky *et al*. highlighted the antineoplastic potential of sertraline, one of the most commonly prescribed antidepressants.^10^ Collectively, these findings offer a plausible biologic explanation for the null or slightly reduced risk observed in our meta-analysis. A similar pattern has been reported for other drug classes; for instance, a systematic review of nonsteroidal anti-inflammatory drugs also found a trend towards reduced skin cancer risk, although it did not reach statistical significance.^28^

Another possible explanation for the reduced skin cancer risk among users of antidepressants found in our study may be related to behavioural patterns associated with depression, such as reduced time spent outdoors, which could result in lower UVR exposure, rather than reflecting a direct pharmacological effect. Some studies have reported that individuals with depression are less likely to engage in outdoor activities, potentially limiting their UVR exposure and, in turn, their risk of skin cancer.^29^

Clinically, our findings suggest that antidepressant use (SSRIs and non-SSRIs) is unlikely to increase the risk of skin cancer. With no statistically significant associations found, there is no basis on which to alter prescribing practices. Clinicians can continue to prescribe antidepressants as indicated, while maintaining standard sun protection and skin cancer screening guidelines, particularly for patients at high baseline risk of developing skin cancer.

The main strength of this study is its inclusive methodology, which encompassed studies from a variety of geographical regions and study designs with no language restriction. However, several limitations must be acknowledged. Most included studies were conducted in Western countries with predominantly White populations, such as the USA and northern European nations. Consequently, the generalizability of the findings to populations with darker skin tones or those living in regions with different UVR exposure patterns may be limited. Another limitation is the grouping of all skin cancer types into a single outcome due to insufficient data on individual cancer subtypes. Therefore, we could not determine associations between antidepressants and specific skin cancer types (e.g. melanoma or NMSC).

Although most studies adjusted for common confounders, factors like skin phototype and polypharmacy were not consistently addressed, introducing potential residual confounding. In the SSRI vs. non-SSRI analysis, confounding by indication is probable, as these drugs are prescribed for different conditions and populations with varying skin cancer risk, which was not accounted for. The absence of MAOIs limits conclusions about the effects of non-SSRIs, as non-SSRIs are mechanistically diverse, and the greatest direct effect on ROS will almost certainly be through MAOIs, rather than the others, because of monoamine oxidase inhibition.^30^ Additionally, some studies had relatively short or poorly defined follow-up and exposure periods, which may limit the ability to detect long-term associations between antidepressant use and skin cancer risk. Also, the substantial heterogeneity observed across studies limits firm conclusions and reflect differences in drug types, exposure definitions, populations, or unmeasured confounders like lifestyle and sun exposure. In our analysis, heterogeneity varied by drug class, suggesting medication type as a contributing factor.

Given the low certainty of evidence, further research is needed to explore the association between antidepressants and skin cancer in greater detail, and to account for important potential confounders. Nationally representative datasets like the Clinical Practice Research Datalink could be used, as they provide a large study population, long-term follow-up and detailed prescribing data. This would enable analyses by antidepressant class and support investigations into specific skin cancer subtypes, helping to inform clinical guidance.

No statistically significant association was found between antidepressant use and skin cancer risk. While a tendency towards reduced risk was observed, the findings are not definitive given the limitations of the included studies. Further large-scale, well-designed studies accounting for important confounders and considering sufficient follow-up and different skin cancer types are needed.

## Supplementary Material

vzag028_Supplementary_Data

## Data Availability

The data underlying this article will be shared on reasonable request to the corresponding author.

## References

[1] Cancer Research UK . Melanoma skin cancer statistics. Available at: https://www.cancerresearchuk.org/health-professional/cancer-statistics/statistics-by-cancer-type/melanoma-skin-cancer#heading-Ze (last accessed 2 September 2024).

[2] Brochez L, Volkmer B, Hoorens I et al Skin cancer in Europe today and challenges for tomorrow. J Eur Acad Dermatology Venereol 2025; 39:272–7. 10.1111/jdv.20368 39377431

[3] George EA, Baranwal N, Kang JH et al Photosensitizing medications and skin cancer: a comprehensive review. Cancers (Basel) 2021; 13:2344. 34066301 10.3390/cancers13102344 PMC8152064

[4] Robinson SN, Zens MS, Perry AE et al Photosensitizing agents and the risk of non-melanoma skin cancer: a population-based case–control study. J Invest Dermatol 2013; 133:1950–5. 23344461 10.1038/jid.2013.33 PMC3655101

[5] Kowalska J, Rok J, Rzepka Z, Wrześniok D. Drug-induced photosensitivity – from light and chemistry to biological reactions and clinical symptoms. Pharmaceuticals 2021; 14:723. 34451820 10.3390/ph14080723 PMC8401619

[6] Brandes LJ, Arron RJ, Bogdanovic RP et al Stimulation of malignant growth in rodents by antidepressant drugs at clinically relevant doses. Cancer Res 1992; 52:3796–800. 1617649

[7] Kubera M, Grygier B, Wrona D et al Stimulatory effect of antidepressant drug pretreatment on progression of B16F10 melanoma in high-active male and female C57BL/6J mice. J Neuroimmunol 2011; 240–241:34–44. 10.1016/j.jneuroim.2011.09.006 22030244

[8] Parker KA, Glaysher S, Hurren J et al The effect of tricyclic antidepressants on cutaneous melanoma cell lines and primary cell cultures. Anticancer Drugs 2012; 23:65–9. 21897201 10.1097/CAD.0b013e32834b1894

[9] Grygier B, Arteta B, Kubera M et al Inhibitory effect of antidepressants on B16F10 melanoma tumor growth. Pharmacol Rep 2013; 65:672–81. 23950590 10.1016/s1734-1140(13)71045-4

[10] Uversky N, Duarte D, Vale N. Antidepressant drug sertraline against human cancer cells. Biomolecules 2022; 12:1513. 36291722 10.3390/biom12101513 PMC9599050

[11] Bogowicz P, Curtis HJ, Walker AJ et al Trends and variation in antidepressant prescribing in English primary care: a retrospective longitudinal study. BJGP Open 2021; 5:1–12. 10.3399/BJGPO.2021.0020 PMC8450889 33985965

[12] Page MJ, McKenzie JE, Bossuyt PM et al The PRISMA 2020 statement: an updated guideline for reporting systematic reviews. BMJ 2021; 372:n71. 33782057 10.1136/bmj.n71 PMC8005924

[13] Higgins JPT, Morgan RL, Rooney AA et al A tool to assess risk of bias in non-randomized follow-up studies of exposure effects (ROBINS-E). Environ Int 2024; 186:108602. 38555664 10.1016/j.envint.2024.108602 PMC11098530

[14] Berge LAM, Andreassen BK, Stenehjem JS et al Use of antidepressants and risk of cutaneous melanoma: a prospective ­registry-based case–control study. Clin Epidemiol 2020; 12:193. 32110111 10.2147/CLEP.S241249 PMC7042562

[15] Berge LAM, Andreassen BK, Stenehjem JS et al Use of immunomodulating drugs and risk of cutaneous melanoma: a nationwide nested case–control study. Clin Epidemiol 2020; 12:1389–401. 33376408 10.2147/CLEP.S269446 PMC7755337

[16] Boursi B, Lurie I, Mamtani R et al Anti-depressant therapy and cancer risk: a nested case–control study. Eur Neuropsychopharmacol 2015; 25:1147–57. 25934397 10.1016/j.euroneuro.2015.04.010

[17] Westerdahl J, Olsson H, Masback A et al Risk of malignant melanoma in relation to drug intake, alcohol, smoking and hormonal factors. Br J Cancer 1996; 73:1126–31. 8624275 10.1038/bjc.1996.216 PMC2074414

[18] Weiss SR, McFarland BH, Burkhart GA Ho PTC. Cancer recurrences and second primary cancers after use of antihistamines or antidepressants. Clin Pharmacol Ther 1998; 63:594–9. 9630832 10.1016/S0009-9236(98)90110-2

[19] Navarro-Bielsa A, Gracia-Cazaña T, Almagro M et al The influence of the exposome in the cutaneous squamous cell carcinoma, a multicenter case–control study. Cancers (Basel) 2023; 15:5376. 38001636 10.3390/cancers15225376 PMC10670280

[20] Almagro M, Navarro-Bielsa A, Gracia-Caza T et al Exposome and basal cell carcinoma: a multicenter case–control study. Int J Dermatol 2024; 63:907–15. 38282244 10.1111/ijd.17026

[21] Navarro-Bielsa A, Gracia-Cazaña T, Almagro M et al [Translated article] Multicenter, prospective, case–control study of exposome in melanoma. Actas Dermosifiliogr 2025; 116:T527–33. 40081473 10.1016/j.ad.2025.03.008

[22] Knuutila JS, Kaijala O, Lehto S et al Clinical risk factors for cutaneous squamous cell carcinoma in patients with actinic keratosis or cutaneous squamous cell carcinoma in situ: a retrospective double-cohort study. Acta Derm Venereol 2024; 104:adv40990. 39601367 10.2340/actadv.v104.40990 PMC11609879

[23] Haukka J, Sankila R, Klaukka T et al Incidence of cancer and antidepressant medication: record linkage study. Int J Cancer 2010; 126:285–96. 19739257 10.1002/ijc.24537

[24] Kirkova M, Tzvetanova E, Vircheva S et al Antioxidant activity of fluoxetine: studies in mice melanoma model. Cell Biochem Funct 2010; 28:497–502. 20803706 10.1002/cbf.1682

[25] García-García ML, Tovilla-Zárate CA, Villar-Soto M et al Fluoxetine modulates the pro-inflammatory process of IL-6, IL-1β and TNF-α levels in individuals with depression: a systematic review and meta-analysis. Psychiatry Res 2022; 307:114317. 34864233 10.1016/j.psychres.2021.114317

[26] Granstein RD, Matsui MS. UV radiation-induced immunosuppression and skin cancer. Cutis 2004; 75:4–9. 15603215

[27] Niu T, Wei Z, Fu J et al Venlafaxine, an anti-depressant drug, induces apoptosis in MV3 human melanoma cells through JNK1/2-Nur77 signaling pathway. Front Pharmacol 2023; 13:1080412. 36686679 10.3389/fphar.2022.1080412 PMC9846499

[28] Ma Y, Yu P, Lin S et al The association between nonsteroidal anti-inflammatory drugs and skin cancer: different responses in American and European populations. Pharmacol Res 2020; 152:104499. 31689521 10.1016/j.phrs.2019.104499

[29] Burns AC, Saxena R, Vetter C et al Time spent in outdoor light is associated with mood, sleep, and circadian rhythm-related outcomes: a cross-sectional and longitudinal study in over 400,000 UK Biobank participants. J Affect Disord 2021; 295:347–52. 34488088 10.1016/j.jad.2021.08.056 PMC8892387

[30] Banerjee C, Tripathy D, Kumar D, Chakraborty J. Monoamine oxidase and neurodegeneration: mechanisms, inhibitors and natural compounds for therapeutic intervention. Neurochem Int 2024; 179:105831. 39128624 10.1016/j.neuint.2024.105831

