# Supplementary material for: Association between prescribed antidepressant medication and skin cancer (melanoma, basal cell carcinoma and cutaneous squamous cell carcinoma) risk: a systematic review and meta-analysis
Source: Skin Health Dis. 2026 Apr 17;6(3):232–41. doi: 10.1093/skinhd/vzag028 (PMC13220031; doi:10.1093/skinhd/vzag028)
Supplement: vzag028_Supplementary_Data [file vzag028_supplementary_data.docx]

**Supplementary materials**

**Appendix 1.** Search strategy

("Skin cancer" OR Melanoma OR "Basal cell carcinoma" OR "Non-melanoma skin cancer" OR "Keratinocyte cancer" OR "Cutaneous squamous cell carcinoma") AND (Antidepressants OR ''Selective serotonin re-uptake inhibitors'' OR SSRIs OR "Serotonin and Noradrenaline Reuptake Inhibitors" OR SNRIs OR ''Monoamine Oxidase Inhibitors'' OR MAOIs OR Amitriptyline OR Fluoxetine OR Mirtazapine OR Citalopram OR Dosulepin OR Nortriptyline OR Sertraline OR Tricyclics OR Vortioxetine OR Fluvoxamine OR Paroxetine OR Escitalopram OR Venlafaxine OR Trazodone)

**Appendix 2. Summary of study exclusions.**

**2a.** Exclusion reasons following full-text screening.

| **Author & Publication year** | **Reason for exclusion** |
| --- | --- |
| Atlantis 2012 | Wrong outcome |
| Blum 2025 | Wrong study design |
| Bosnak 2018 | Wrong study design |
| Boursi 2017 | Wrong outcome |
| Gau 2008 | Wrong publication type |
| Iwagami 2020 | Wrong exposure |
| Onasanya 2021 | Wrong outcome |
| Robinson 2014 | Wrong exposure |
| Rosales 2018 | Wrong exposure |
| Sedgh 2021 | Wrong exposure |
| Steinhausen 2014 | Not adult |
| Tchernev 2023 | Wrong study design |
| Wang 2021 | Wrong population |

**2b.** Exclusion reasons for studies not included in the meta-analysis.

| **Author & Publication year** | **Reason for exclusion** |
| --- | --- |
| Bielsa 2023 | Data for antidepressants and hypnotics were combined; antidepressant-specific data not available. |
| Bielsa 2024 | Data for antidepressants and hypnotics were combined; antidepressant-specific data not available. |
| Bielsa 2025 | Data for antidepressants and hypnotics were combined; antidepressant-specific data not available. |
| Knuutila 2024 | Included patients with precancerous lesions (actinic keratosis and SCC in situ). |
| Weiss 1998 | Data for antidepressants and antihistamines were combined; antidepressant-specific data not available. |

**Appendix 3. Additional study characteristics and methodological details.**

| **Case-control studies** | | | | | | | |
| --- | --- | --- | --- | --- | --- | --- | --- |
| **Author & publication year** | **Age (as reported)** | | **Exposure duration** | **Follow-up period** | **Exposure assessment method** | **Control type** | **Matching variables (controls)** |
|  | **Cases** | **Controls** |  |  |  |  |  |
| Berge et al (2020a) | <50 = 26.1%  50–69 = 46.4%  ≥70 = 27.5%^[[1]](#footnote-1)^ | <50 = 25.6% 50–69 = 46.6%  ≥70 = 27.8%a | ≤5 years; >5 years | NR | Prescription registry | Risk-set, randomised | Sex, year of birth |
| Berge et al (2020b) | <50 = 26.1%  50–69 = 46.4% ≥70 = 27.5%^a^ | <50 = 25.6% 50–69 = 46.6% ≥70 = 27.8%^a^ | ≤5 years; >5 years | NR | Prescription registry | Risk-set, randomised | Sex, year of birth |
| Bielsa et al (2023) | 76.46±10.11^[[2]](#footnote-2)^ | 55.77 ± 15^b^ | NR | NR | Self-reported questionnaire | Companions, non-randomised | Age, sex |
| Bielsa et al (2024) | 66.9±12.6^b^ | 55.7 ± 15^b^ | NR | NR | Self-reported questionnaire | Companions, non-randomised | Age, sex |
| Bielsa et al (2025) | 56.0±15.1^b^ | 55.7 ± 15^b^ | NR | NR | Self-reported questionnaire | Companions, non-randomised | Age, sex |
| Boursi et al (2015) | 60.4±16.6^b^ | 60.2 ± 16.6^b^ | ≤1 year; 1–3 years; >3 years | NR | Electronic prescription records | Risk-set, non-randomised | age, sex, practice site, follow-up, calendar period |
| Weiss et al (1998) | 20–39: 7.0%  40–59: 32.0%  60–79: 51.4%  ≥80: 9.6%^a^ | | NR | 6 months after treatment/diagnosis | Electronic prescription records | Risk-set, randomised | NR |
| Westerdahl et al (1995) | 15-75 years^[[3]](#footnote-3)^ | | NR | NR | self-reported questionnaire | Population-based, randomised | sex, age, parish |
| **Cohort studies** | | | | | | | |
| Haukka et al (2009) | Females: 48 (35–58); Males: 45.8 (34–56)^b^ | | NR | Mean 4.01 years | Prescription registry | Non-users | Birth year, sex, region |
| Knuutila et al (2024) | 73.5 ± 10.5^b^ | | ≥1 year | 5 years | Patient records | Non-users | NR |

Abbreviations: NR = Not reported by study authors (data unavailable).

**Appendix 4.** Risk of bias results (ROBINS-E tool).

| **Author & Year** | **Berge (2020a)** | **Berge (2020b)** | **Bielsa (2023)** | **Bielsa (2024)** | **Bielsa (2025)** | **Boursi (2015)** | **Haukka (2009)** | **Knuutila (2024)** | **Weiss (1998)** | **Westerdahl (1995)** |
| --- | --- | --- | --- | --- | --- | --- | --- | --- | --- | --- |
| **Bias due to confounding** | Moderate | Moderate | Moderate | Moderate | Moderate | Moderate | Moderate | Moderate | High | High |
| **Bias in selection of participants into the study** | Low | Moderate | High | High | High | Low | Moderate | Moderate | Moderate | Low |
| **Bias in classification of exposure** | Low | Low | Moderate | Moderate | Moderate | Low | Low | Unclear | Low | Low |
| **Bias due to deviations from intended exposures** | Low | Low | High | High | High | Low | Moderate | Unclear | Low | Low |
| **Bias due to missing data** | Unclear | Moderate | Unclear | Unclear | Unclear | Moderate | Moderate | High | Moderate | Low |
| **Bias in measurement of outcomes** | Low | Low | Low | Low | Low | Low | Low | Low | Low | Low |
| **Bias in selection of the reported result** | Low | Moderate | Low | Low | Low | Low | Low | Low | Low | Low |
| **Overall risk of bias** | Moderate | Moderate | High | High | High | Moderate | Moderate | High | High | High |

1. Age reported as categorical distribution. [↑](#footnote-ref-1)
2. Age reported as mean ± standard deviation. [↑](#footnote-ref-2)
3. Only inclusion age range reported by the study [↑](#footnote-ref-3)
